# Reticulon-3 modulates the incorporation of replication competent hepatitis C virus molecules for release inside infectious exosomes

**DOI:** 10.1371/journal.pone.0239153

**Published:** 2020-09-17

**Authors:** Jingjing Li, Ebtisam Abosmaha, Carla S. Coffin, Patrick Labonté, Terence Ndonyi Bukong

**Affiliations:** 1 INRS-Institut Armand-Frappier, Institut National de la Recherche Scientifique, Laval, Québec, Canada; 2 Department of Medicine, University of Calgary, Calgary, Alberta, Canada; CIMA, SPAIN

## Abstract

**Background:**

Cell released microvesicles specifically, exosomes, play an important role in mediating immunologic escape, treatment resistance, and disease persistence of Hepatitis C virus (HCV) infection. Reports on the molecular compositions of exosomes released by cells under diverse conditions, especially during viral infections, suggest that their cargo contents are not randomly loaded. However, the precise molecular mechanisms directing the selective cargo sorting and loading inside infectious viral exosomes remains elusive.

**Aim:**

To decipher the role of Reticulon 3 (RTN3) in the selective molecular cargo sorting and loading inside infectious viral exosomes during HCV infection.

**Methods:**

We used Huh7 cells–JFH1 HCV infection and HCV Full-Length (FL) replicon systems. Additionally, we analyzed human liver and serum exosome samples from healthy and treatment naïve HCV infected individuals. Our experiments made use of molecular biology and immunology techniques.

**Results:**

HCV infection (JFH1-Huh7 or HCV-FL replicon cells) was associated with increased RTN3L&S isoforms expression in cells and cell released exosomes. Accordingly, increased expression of RTN3L&S was observed in liver and serum exosome samples of HCV infected individuals compared to healthy controls. RNA-ChIP analysis revealed that RTN3L&S interacted with dsHCV RNA. Lentiviral CRISPR/Cas9 mediated knockdown (KD) of RTN3 and plasmid overexpression (OE) of wild type, C- and N-terminal deletion mutants of RTN3L&S in HCV- infected Huh7 cells differentially impacted the cellular release of infectious viral exosomes. RTN3L&S KD significantly decreased, while RTN3S OE significantly increased the number of Huh7 cell-released infectious exosomes. The C-terminal domain of RTN3 interacted with and modulated the loading of dsHCV RNA inside infectious exosomes. Antiviral treatment of HCV infected Huh7 cells reduced virus-induced RTN3L&S expression and attenuated the release of infectious exosomes.

**Conclusion:**

RTN3 constitutes a novel regulator and a potential therapeutic target that mediates the specific loading of infectious viral exosomes.

## Introduction

Hepatitis C virus (HCV) infection is an important cause of morbidity and mortality globally. HCV is an envelope positive-sense single-stranded RNA Flavivirus with a genome size of approximately 9.6kb [1]. The HCV genome contains an open reading frame (ORF) encoding a single polyprotein which is cleaved by cellular and viral enzymes into ten mature proteins [1]. There is currently no effective HCV vaccine and the World Health Organization estimates that there are over 71 million [2] individuals worldwide with active infection. If untreated, approximately 70–80% of HCV infected individuals will develop complications with progressive liver fibrosis, cirrhosis, and hepatocellular carcinoma [3, 4]. The use of pan-genotypic direct-acting antiviral (DAAs) regimens is curative in >95% of HCV infected individuals [5]. However, access to diagnosis and treatment in some countries is very limited and resistance to some DAA treatment regimens is continuously being reported [6–8]. The propensity of HCV to establish persistent infection stems from multiple remarkable strategies by the virus to evade host immune and therapeutic strategies [9, 10]. In 2013–2014 groundbreaking reports including our study revealed that viruses can hijack cell-released extracellular vesicles (EVs), precisely, exosomes, to evade host immune and therapeutic strategies leading to persistent infection [11, 12]. Specifically, these studies revealed that exosomes can harbor replication-competent viral material and can bypass classical receptor-mediated viral entry mechanisms to facilitate active viral infection of naïve cells [11, 12]. Strikingly, recent scientific reports have also implicated exosomes in the pathomechanism of several viral infections including HBV [13], the Human Immunodeficiency Virus (HIV) [14], Human T-cell Lymphotropic Virus (HTLV) [15], Ebola Virus [16] and Zika Virus [17].

Exosomes are cell-derived microvesicles that are continuously produced by almost all cell types into the extracellular space and range in size from 30 to 150 nm. The molecular composition of exosomes most often reflects the physiological/pathological state of the cells they originate from [18]. In addition to their pathogenic role, exosomes also carry out important cellular communication functions by interacting and /or transferring their cargo contents to target recipient cells altering their function in precise ways [19]. Even when released by the same cell, each exosome is composed of a specific repertoire of proteins, lipids, and nucleic acids while others are excluded [20]. These observations suggest that the molecular cargo found inside a specific exosome is not randomly loaded. Numerous mechanisms have recently been proposed on how specific cellular molecules by utilizing specialized cellular mechanisms can modulate the molecular composition of exosomes in both normal, stress, and infection conditions [21]. Studies have proposed that exosomal protein composition can be controlled by endosomal complex required for transport (ESCRT)-dependent and -independent mechanisms [22]. Notwithstanding, most described mechanisms seem to act differently depending on the cell type and stimuli/infection which ultimately results in the production of different subsets of exosomes even by the same cell. While exosome biogenesis was suggested to originate from multivesicular bodies (MVB) [20], observations of different types of exosomes from the same cell suggest the possible existence of different MVB subsets. Taken together, the mechanisms by which specific cellular molecules are selectively loaded inside exosomes while others are excluded especially during viral infections remain poorly understood.

Here, we explore for the first time the role of cellular Reticulons (RTNs), specifically RTN3, in modulating the specific incorporation of host and replication-competent viral molecules for release inside exosomes. Reticulons (RTNs) are a large family of evolutionarily conserved proteins predominantly located at the endoplasmic reticulum (ER) of cells. They are most often associated with membrane morphogenesis, intracellular trafficking, and microvesicle formation [23, 24]. This family of proteins contains four main gene products (RTN1, RTN2, RTN3, and RTN4) [23]. Genes encoding for reticulons contain many introns and exons, and most are alternatively spliced into multiple protein isoforms [25]. Morphologically, RTNs contain a highly conserved C-terminal Reticulon Homology Domain (RHD) and an N-terminal domain which is highly variable [23, 24]. RTN proteins are mostly enriched in the nervous tissue, however, RTN3 and RTN4 are expressed ubiquitously [23]. Primarily RTNs have been associated with promoting membrane curvature development, nuclear pore complex formation, vesicle maturation, autophagy, and inflammatory functions [23, 26, 27]. In the context of viral infections, RTNs have been associated with the replication of single-stranded RNA viruses and membrane trafficking of early secretory proteins [23, 28]. Recent reports have revealed that RTN3 can mediate viral remodeling of host cell membranes and the stabilization of viral proteins within the endoplasmic reticulum during flavivirus replication [28]. Furthermore, RTN3 has been associated with direct modulatory role during HCV viral replication [29, 30]. However, the role of RTN3 in modulating the selective cargo sorting associated with infectious viral exosomes release from HCV infected cells has not been evaluated.

Here, we reveal RTN3 as a novel regulator that mediates the specific loading of infectious cell-released viral exosomes. Our observations also suggest that RTN3 represents a novel infection biomarker and therapeutic target that can be exploited to prevent the cellular release of infectious viral exosomes associated with HCV infection.

## Material and methods

### Cell lines and hepatitis C virus

Huh7 cells (a gift from Dr. Charlie Rice, Rockefeller University, New York) and HCV FL replicon [HCV genotype 1b] (a gift from Takaji Wakita) were cultured as previously described [11] with slight modification, using exosome depleted FBS (System Bioscience cat. #Exo-FBS-50A-1). Infectious HCV genotype 2a (clone JFH1) virus was generated as previously described [31, 32].

### Exosome isolation from cell culture supernatants and human serum samples

Cell culture supernatants of Huh7, JFH1 infected Huh7 and HCV FL replicon cells were collected and centrifuged at 800× g for 5 minutes to remove cell debris. To concentrate microvesicles in cell culture supernatants, cleared supernatants were then transferred into Amicon Ultra-15 Centrifugal Filter Unit with Ultracel-100 membrane (Millipore, cat. #UFC910024) and followed by series of centrifugations at 3,500× g for 20 minutes. Concentrated culture supernatants were mixed with the appropriate volume (50μl—150μl) of Exoquick (System Bioscience cat. #EXOQ5A-1) according to the manufacturers’ specifications and centrifuged at 6000×g for 20min. The exosome pellet was washed 2 times with PBS by centrifugation at 800x g at 4°C for 5 minutes and re-suspended in 1X phosphate buffer saline (PBS). For JFH1 and serum exosomes from human samples, further positive anti-CD63 immuno-selection was performed as described previously [11].

### Transfections of siRNA and over-expression plasmids

Cellular transfections were done using Lipofectamine 2000 (Invitrogen, Carlsbad, CA) according to the manufacturer’s protocol. Small interfering RNA (siRNA) targeting Reticulon (RTN3) or control (scrambled) (Life Technologies). Overexpression plasmid with a flag-CMV-2 backbone was used for expressing RTN3S full-length (FL), N-terminally or C-terminally truncated RTN3s mutants (i.e., ΔN11, ΔN35, ΔN45, and ΔC36) (gift provided Prof. Mitsuo Tagaya, Tokyo University of Pharmacy and Life Science) [24], and pMRX-INU-FLAG-RTN3L (gift from Noboru Mizushima, Addgene plasmid # 128264; http://n2t.net/addgene:128264; RRID: Addgene_128264) [33].

### Lentiviral CRISPR-Cas9 knockdown, transfections, and Co-culture experiments

Specific Reticulon 3 target guide RNA sequences for CRISPR-Cas9 were designed using the website (http://www.e-crisp.org/E-CRISP/). Specific oligonucleotide primer pairs were used to amplify the long and short isoforms of human Reticulon 3: RTN3L Forward: 5'-CACCGCCATGTGTTAGGGAGCCAGCCT-3'; RTN3L Reverse complement: 5'-AAACAGGCTGGCTCCCTAACACATGGC-3' and RTN3S Forward:5'-CACCGGGAGATGGAATGGGACTGAG-3', and RTN3S Reverse complement: 5'-AAACCTCAGTCCCATTCCATCTCCC-3'. The complementary oligonucleotides for guide RNAs (gRNAs) were annealed and cloned into the LentiCrispr V2 vector [LentiCrispr, a gift from Feng Zhang; Addgene plasmid # 52961; http://n2t.net/addgene:52961; RRID: Addgene_52961)] [34]. The human GeCKOv2 CRISPR knockout pooled library was a gift from Feng Zhang (Addgene catalog #1000000048) [34]. Hemagglutinin (HA) lentivirus vector was used as a control. The LentiCrispr V2 vector was transfected into HEK-293T cells using Lipofectamine 2000 and media exchanged after 12h. The lentivirus was harvested from the cell culture media after three days, and viral particles were concentrated by ultracentrifugation. Huh7-JFH1 infected and FL Replicon cells were co-cultured with the RTN3L&S CRISPR-Cas9 lentiviruses or the HA lentivirus control. Cellular RTN3L&S expression in target cells was analyzed by western blotting.

### Real-time quantitative PCR for detection of HCV RNA from cells and exosomes

Total RNA was extracted by initially lysing cells with Trizol (Invitrogen, CA), and purified using RNeasy micro kit (cat. #74004, Qiagen) according to the manufacturers’ instructions. Briefly, 1μg RNA was subjected to reverse transcription using the iScript reverse transcription supermix (Bio-Rad, California, United States). The obtained cDNAs were used for qPCR using a Bio-Rad CFX96 system with SYBR green. The target primers/reaction conditions used methods described previously [35]. The comparative delta-delta ct method was used to analyze specific genes using 18s RNA as the normalizing gene, similar to previous studies [11, 36].

### Western blotting to detect specific proteins

Total cellular proteins from Huh7.0, JFH1-infected Huh7.0, and HCV FL replicon cells were extracted using RIPA buffer (cat. #BP-115, Boston Bioproducts) as previously described. Proteins resolution was done on an 8–15% gel SDS-PAGE denaturing gel. Resolved proteins in acrylamide gels were transferred onto Polyvinylidene fluoride (PVDF) membranes and membranes were blocked in PBS containing 5% dry milk for 1h, then probed with specific primary antibodies in the recommended dilutions at 4°C for overnight. The following primary antibodies were used: anti-HCV NS3 (Abcam cat. #ab13830 and cat. #ab65407); anti-HCV NS5A (Abcam cat. # ab13833 and BioFront cat. # HCV-2F6); anti-HSP90 (Abcam cat. # ab13492); anti-FLAG (Abcam cat. #ab1162); anti-CD63 (Abcam cat. #ab8219 and Santa Cruz Biotechnology cat. #sc-15363); RTN3 antibody (Abcam cat. Ab68328 and Thermofisher cat. # PA5-53360); TSG101 (Abcam cat. # ab125011); normal rabbit IgG-AC antibody (Santa Cruz Biotechnology cat. # sc-2345); anti-beta actin [Ac-15] (Abcam, cat. #ab6276). Appropriate horseradish peroxidase (HRP)-conjugated secondary antibodies (Santa Cruz Biotechnology cat. # sc-2004 and sc-2005) and clarity TM Western ECL blotting substrate (Bio-Rad) was used for visualization with the ChemiDoc XRS+ system (Bio-Rad, California, United States).

### Exosome quantification in HCV infected vs. control in vitro and Ex-Vivo clinical samples

Quantification of cell released microvesicles were done using Nanoparticle Tracking Analysis (NTA) with NanoSight NS300 (Malvern, UK) and NanoSight NTA software v3.2. Briefly, cell culture supernatants and human serum samples were pre-cleared to remove any cellular debris by centrifugation at 5000 Revolutions Per Minute (RPM) for 5 minutes. The cleared cell culture supernatants and serum samples were filtered through a 0.22 μM membrane into sterile tubes. The quantification of exosomes was done using the NTA measurement system as follows: 25°C, 25 frames per second, 3 measurements per sample.

### RNA chromatin immunoprecipitation (ChIP) and co-immunoprecipitation analysis

Cell samples were fixed at room temperature with 4% formaldehyde buffered saline. Cells were subsequently lysed in SDS ChIP lysis buffer (Millipore cat. # 20–163) supplemented with protease and RNase inhibitor. Total cellular proteins were pre-cleared with protein G beads. 100 μg of total protein was incubated with anti-dsRNA, RTN3S, and RTN3L antibodies. Immunoprecipitation was performed overnight at 4°C using 10 μg/ml primary antibody and normal rabbit/mouse IgG (Santa Cruz cat #sc-3877 and sc-69786) non-specific antibody serving as IP control. A mixture of Protein A/G PLUS-Agarose beads (Santa Cruz cat. #sc-2003) was added, and the incubation was continued for an additional 60 minutes. The samples were washed with SDS ChIP lysis buffer supplemented with protease inhibitor and RNase inhibitor. The immunoprecipitants (protein-RNA complexes) were either used for Western blot analysis or RNA purification using the Trizol reagent and RNeasy kit. Purification of RNA included spiking all samples with 1uL of cel39RNA before total RNA extractions following RTN3L and RTN3S pull-downs. Negative -sense HCV RNA and miR122 quantification were done using RT-qPCR with CFX Connect Real-Time PCR Detection System (Philadelphia, USA), using methods described previously. RT-qPCR data were normalized to miR Cel39 and fold change was calculated using the delta-delta ct method as previously described [11].

### Human samples and ethics statement

Human serum samples were collected at the University of Calgary Liver Unit under an approved ethics protocol for use of human samples for research [Conjoint Ethics Research Board (CHREB) ID: REB14-1965_REN1]. All healthy control subjects had no evidence of systemic disease, HCV infection, or other liver diseases. All subjects who donated samples for this project provided signed written informed consent. Human liver biopsy specimens were obtained from the liver tissue cell distribution system (LTCDS), [Minneapolis, Minnesota] [Pittsburgh, Pennsylvania] [Richmond, Virginia], which was funded by NIH contract # N01-DK-7-004/HHSN26700700004C.

### Data analysis

Data were presented as mean +SEM. Statistical analysis was carried out by using the Mann-Whitney U test on GraphPad Prism version 8.1.2. All data presented are representative of at least 3–4 independent repeat experiments. A p-value of <0.05 was considered significant.

## Results

### HCV infection is associated with increased Reticulon-3 expression in Huh7 cells and exosomes

**Fig 1:**
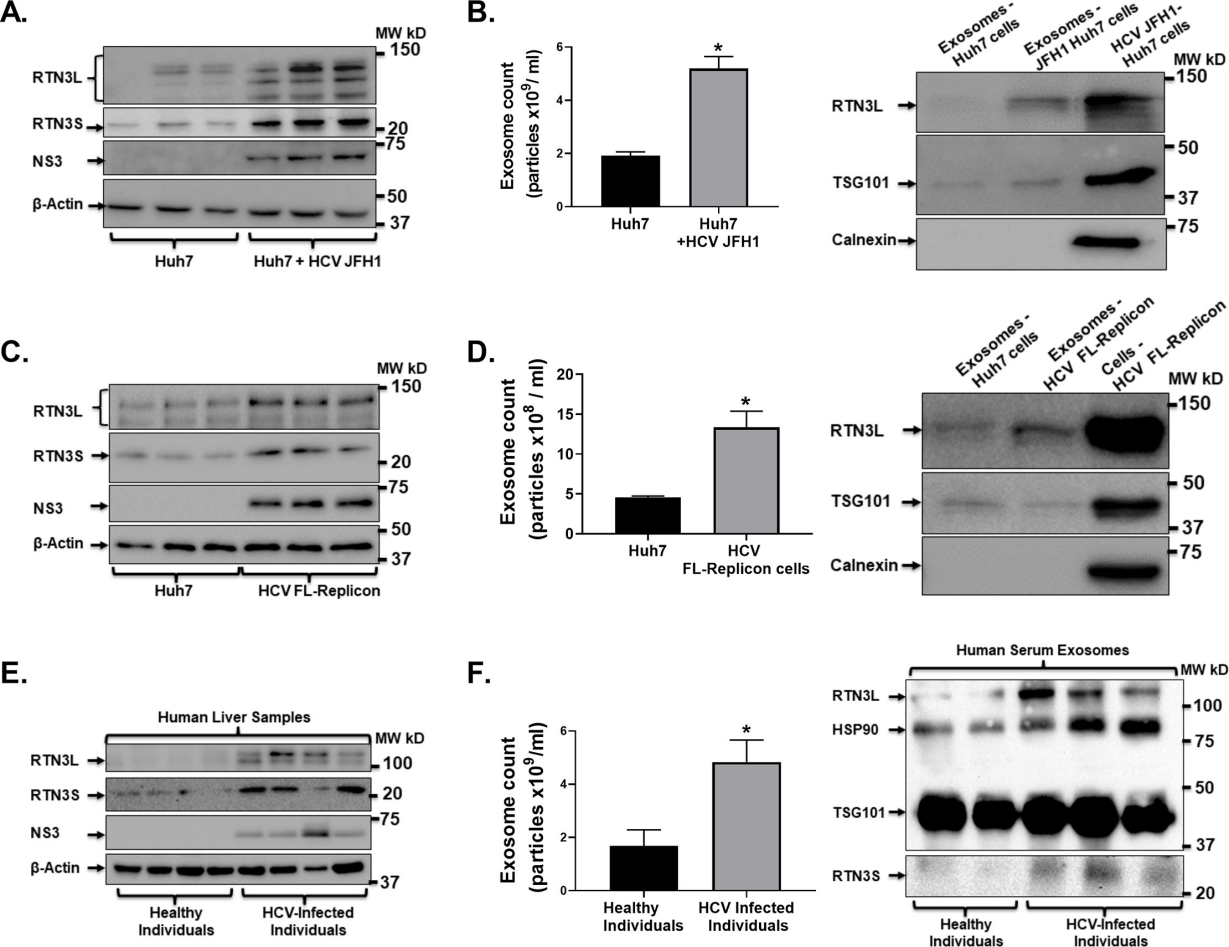
HCV infection induces increased RTN3L&S expression in Huh7 cells and cell released exosomes. (**A**) Total cell proteins were extracted from Huh7 and HCV JFH1 infected Huh7 cells and analyzed by western blotting probing for RTN3L&S and HCV NS3 with β-actin serving as a control for equal protein loading. (**B-Left panel**) Cell released exosomes from Huh7.0 and JFH1 infected Huh7 cells and quantified by NanoSight NS300. (**B-Right panel**) Total proteins were extracted from cell released exosomes in cell culture media of Huh7.0 cells and HCV JFH1 infected Huh7.0 cells, with total cell proteins from HCV JFH1 infected Huh7.0 cells serving as a positive control. Extracted total proteins from exosomes were analyzed by western blotting probing for RTN3L, TSG101, and Calnexin. (**C-Left panel**) Total cell proteins were extracted from Huh7.0 and HCV FL-Replicon cells and analyzed by western blotting probing for RTN3L&S and HCV NS3 with β-actin serving as a control for equal protein loading. (**C-Right panel**) Cell released exosomes from Huh7.0 and HCV FL-Replicon cells were quantified by NanoSight NS300. (**D-Right panel**) Total proteins were extracted from cell released exosomes in cell culture media from Huh7.0 cells and HCV FL-Replicon cells with total cell protein from HCV FL-Replicon cells serving as a positive control. (**D-Left panel**) Total protein was then analyzed by western blotting probing for RTN3L, TSG101, and Calnexin. (**E-Left panel**) Total cell proteins were extracted from liver samples from healthy and HCV infected individuals and analyzed by western blotting probing for RTN3L&S and HCV NS3 with β-actin serving as a control for equal protein loading. (**F-Left panel**) Serum exosomes were isolated from healthy individuals and HCV infected individuals were quantified by NanoSight NS300. (**F-Right panel**) Total proteins were extracted from human serum exosomes as indicated and analyzed for RTN3L&S, HSP90, and TSG101. (In-vitro data is representative of 3 independently repeated experiments. Human tissue samples are representative of 5 healthy and 8 HCV infected individuals. *p<0.05 was considered significant by Mann–Whitney U test.

A recent report revealed increased intrahepatic RTN3 expression which correlated with higher HCV genotype 1 RNA levels [37]. Further, conflicting reports have indicated that RTN3 knockdown in cells can lead to either an increase [29] or no change [30] in intracellular HCV RNA levels. Also, RTN3 has been shown to modulate intercellular molecular trafficking and linked to microvesicles biogenesis and secretion by cells [24, 27, 38]. Total protein was extracted from Huh7 and HCV JFH1 infected Huh7 cells. We found that HCV JFH1 infection of Huh7 cells was associated with increased RTN3L&S isoform proteins compared to control (uninfected) cells by western blotting analysis (**Fig 1A**). Similar to our previous report [11], we found that HCV JFH1 infection of Huh7 cells was associated with the increased cellular release of exosomes (**Fig 1B**). We used exosome isolation and NanoSight NS300 quantification methods as described previously [11]. We found that cell released exosomes from HCV JFH1 infected Huh7 cells were significantly enriched with RTN3L, based on western blot analysis (**Fig 1B**). We did not detect Calnexin protein in our analyzed exosome samples (**Fig 1B**), suggesting that our methodology (as described previously [11]), successfully excluded microsomal or cellular debris contamination [39]. Next, we evaluated whether RTN3L&S expression in HCV FL-replicon cell-released infectious exosomes but not free virions. By western blotting analysis, we found increased expression of RTN3L&S isoforms in HCV FL-replicon cells compared to parent Huh7 control cell line (**Fig 1B**). Additionally, compared to control (uninfected) cells, HCV FL-replicon cells released significantly more exosomes (**Fig 1C**). Similar to JFH1 infection (**Fig 1B**), the released exosomes from HCV FL-replicon cells was associated with increased RTN3L protein expression (**Fig 1D**).

We used human clinical samples from healthy and HCV infected individuals (**Tables 1 and 2**) to enhance the translational relevance of our study and the potential role of RTN3 in HCV infection. We found that HCV infection was associated with increased hepatic RTN3L&S protein expression (**Fig 1D**). Specifically, we found by western blotting analysis an increase in RTN3L&S protein expression in HCV infected individuals compared to healthy controls (**Fig 1E**). We also detected, as previously reported [11], that serum from persons with HCV infection showed significantly increased exosomes compared to control subjects (**Fig 1F**). Strikingly, we found by western blotting analysis that serum exosomes samples of HCV infected individuals were also enriched with RTN3L&S (**Fig 1F**), and HSP90 (**Fig 1F**), consistent with our previous observations [11]. We used β-actin as a loading control for total cellular protein and TSG101 as a loading control for exosomal analysis by western blotting (**Fig 1**).

**Table 1:** Clinical parameters of serum from HCV infected individuals.

Parameters	Distribution
Gender: Male/Female	4/3
Age	50.2 (±2.89)
Genotypes	1, 1a, 2, 4
ALT (IU/mL)	110.6 (±31.3)
Treatment Status	Untreated or Harvoni Treatment Failure

5 Samples from healthy uninfected subjects served as controls.

**Table 2:** Clinical parameters for HCV human liver samples.

Parameters	Distribution
Gender: Male/Female	5/6
Age	56.4 (±2.57)
Genotypes	1, 2 and 4
AST (IU/mL)	99.36 (±21.0)
Albumin (g/dL)	2.76 (±0.13)
Alkaline Phosphatase (U/L)	124.18 (±15.80)
Creatinine (mg/dL)	1.64 (±0.40)
Total Bilirubin	8.27 (±3.56)
Prothrombin Time (INR)	2.56 (±0.20)
Histopathology Report Summary	Chronic inflammation with Cirrhosis or end-stage Cirrhosis secondary to active Hepatitis C virus infection.

10 Samples from uninfected donors with intracranial hemorrhage, cerebrovascular accident, and accident victims served as controls.

### Reticulon-3 interacts with and is in complex with dsHCV RNA and HCV NS3

**Fig 2:**
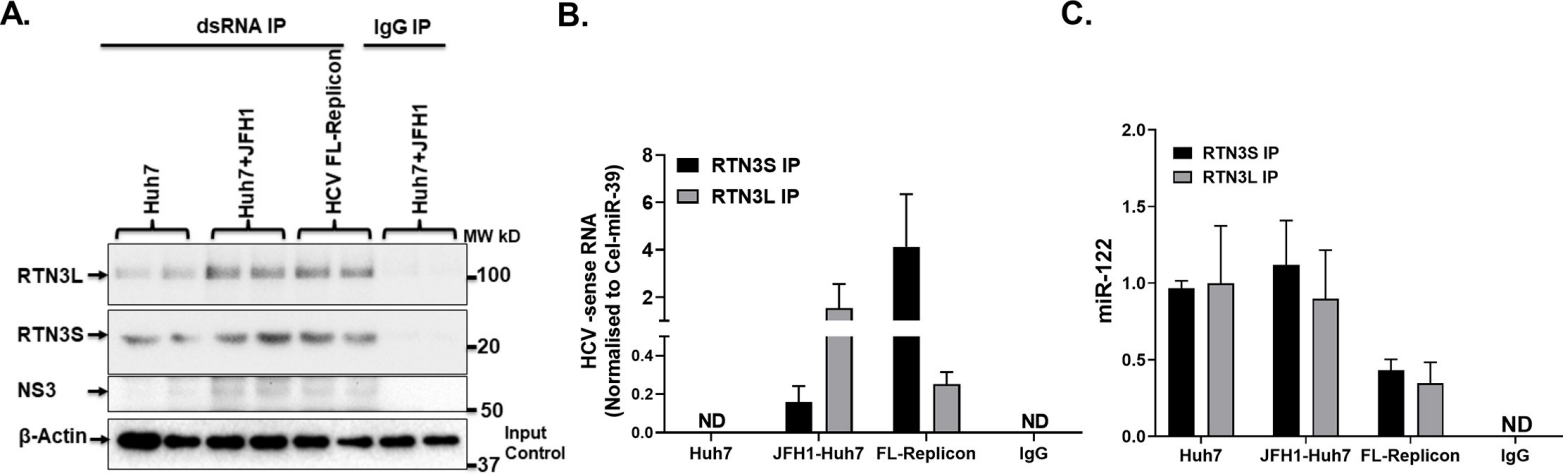
RTN3L&S interact with double-stranded viral RNA in HCV infected Huh7 cells. (**A**) RNA ChIP analysis was performed from cell lysates from Huh7.0, Huh7.0+JFH-1 infected, and HCV FL-Replicon cells. Immuno-precipitations of cell lysates were performed using specific dsRNA and non-specific IgG antibodies using protein A/G pulldown. (**A**) Pull-down proteins interacting with dsRNA were probed by western blotting targeting for RTN3L&S and HCV NS3. Input total lysates were subjected to western blot analysis and probed for β-Actin. (**B**) Total RNA was isolated from Immuno-precipitations of cell lysates following the pulldown of RTN3L&S and non-specific IgG antibodies. Total RNA extracted was subjected to RT-qPCR targeting (**B**) negative-sense HCV RNA and (**C**) miR-122. miR-Cel39 RNA served as an exogenous loading control. Data is representative of 3 independently repeat experiments with p<0.05 was considered significant by the Mann–Whitney U test.

RTN3 has been shown to interact with HCV NS4B required for the formation of the membranous web that is obligatory for HCV replication [29, 40, 41]. However, the interaction between RTN3 and dsHCV RNA the predominant form in human liver and interferon‐treated cells [42] has not been demonstrated. Given our interest in deciphering the role of RTN3 in infectious exosomes loading, we assessed if RTN3 interacted with dsHCV RNA. Our RNA ChIP analysis revealed that dsHCV RNA interacts with RTN3L&S which is also in complex with HCV NS3 (**Fig 2A**). However, RTN3L&S isoform showed differential levels of interactions with dsRNA (**Fig 2B**) and miR-122 (**Fig 2C**) in HCV JFH1 infected Huh7 and HCV FL-replicon cells. While dsHCV RNA interacted more with RTN3L in JFH1 infected Huh7 cells, interactions in HCV FL-replicon cells involved mainly RTN3S (**Fig 2C**). miR-122 interaction with RTN3L&S was also much lower in FL-replicon cells compared to uninfected and JFH-1 infected Huh7 cells (**Fig 2C**).

### Knockdown of Reticulon-3 impacted the cellular release of replication-competent viral-exosomes from HCV infected cells

**Fig 3:**
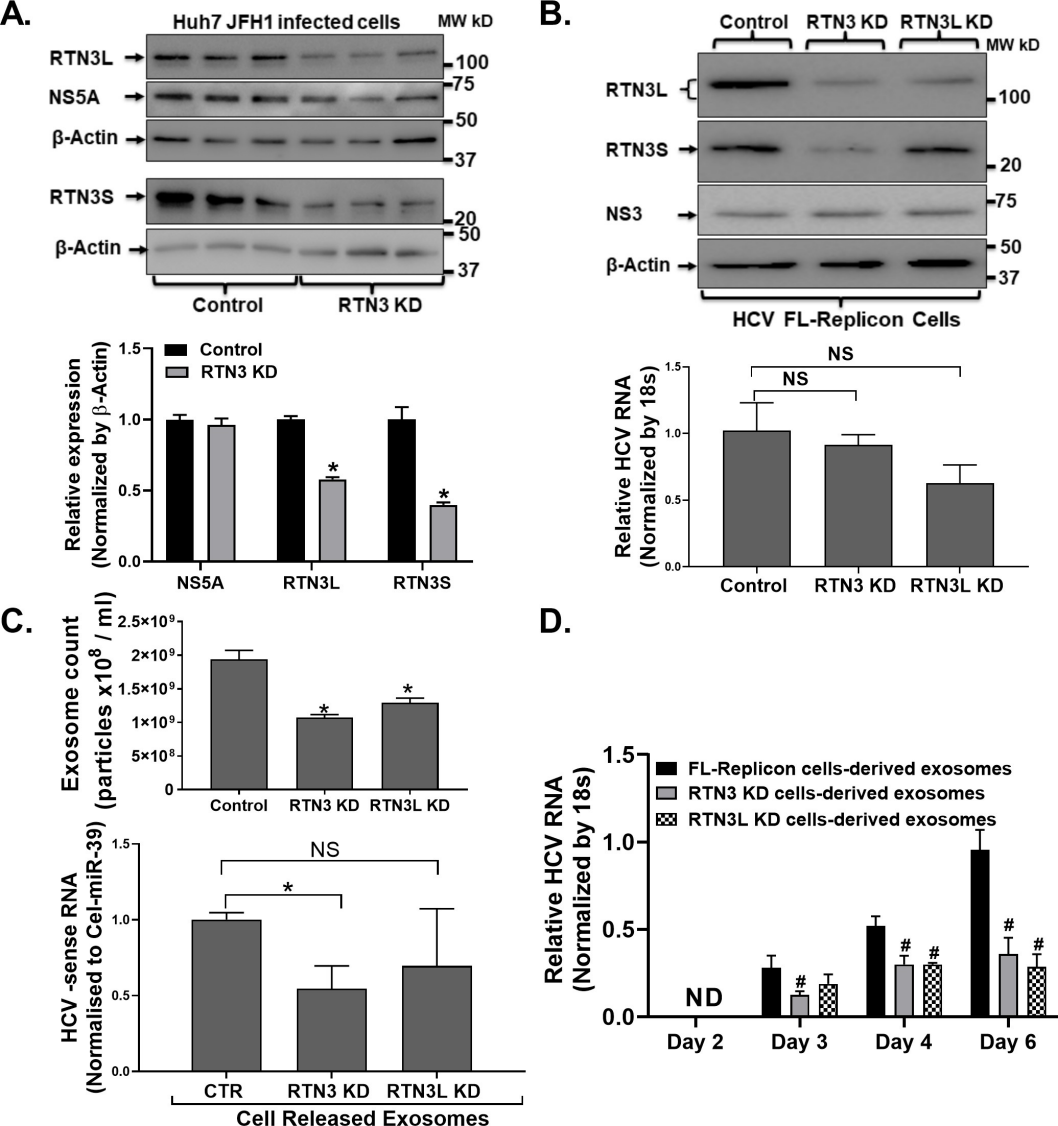
RTN3 modulates the loading of infectious viral molecules inside exosomes. (**A&B**) Reticulon 3L&S (RTN3L&S) was knockdown using specific siRNA(**A**) and LentiCRISPR lentiviral CRISPR/Cas9 (**B**) methods in HCV FL-Replicon cells alongside appropriate control as indicated. 48h post RTN3 knockdown, cell culture supernatants, and cells were harvested. Total proteins extracted from cells were subjected to western blotting probing for RTN3L&S, NS5A, and NS3 with β-actin serving as an equal loading control. (**C**) NanoSight NS300 was used to quantify cell released exosomes in cell culture supernatants. (**D**) Cell released exosomes from LentiCRISPR lentiviral CRISPR/Cas9 knockdown of RTN3L&S conditioning and was co-cultured with naïve Huh7.0 cells (5k Exosomes: 1 Huh7.0 cell) over 72h. Total RNA was then extracted from cells and analyzed by RT-qPCR for HCV RNA using 18s as a housekeeping gene. Data is representative of 3 independently repeat experiments with */^**#**^p<0.05 was considered significant by Mann–Whitney U test.

The presence of infectious viral material inside exosomes is a classical feature of hepatitis C, well recognized in human infection and cell culture systems [11]. How replication-competent viral material gets loaded into exosomes in general currently remains ill-defined. To address this knowledge gap, we investigated whether any relationship existed between infectious exosome generation and RTN3. First, we transiently knockdown RTN3L&S isoforms by siRNA methods in JFH1 infected Huh7 cells (**Fig 3A**). We observed by western blotting analysis that RTN3L&S knockdown in HCV JFH1 infected Huh7 cells did not impact HCV NS5A protein levels (**Fig 3A**) Similarly, RTN3L&S knockdown in HCV FL-replicon cells using a lentiviral CRISPR/Cas9 did not impact HCV NS3 protein expression (**Fig 3B-Upper panel**). However, the was a modest but not statistically significant reduction of HCV RNA in FL-replicon cells following RTN3L&S knockdown (**Fig 3B- Lower panel**). These observations suggested that knockdown of RTN3L&S did not impact viral protein translation but might modestly impact cellular viral RNA replication. Strikingly, both RTN3L&S knockdowns were associated with a significant reduction of cell released exosomes from HCV FL-replicon cells (**Fig 3C**). Also, knockdown of RTN3L&S significantly reduced the amount of HCV RNA loaded inside cell released exosomes (3C-Upper panel). Notably, Knockdown of RTN3L in HCV infected cells resulted in a modest but not statistically significant enrichment of negative-sense HCV RNA in cell released exosomes suggesting possible compensatory effects of RTN3S (**Fig 3C-Lower panel**). Further, the co-culture of cell released exosomes from RTN3L&S knockdown cells with naïve uninfected Huh7 cells was associated with less robust infection as revealed by HCV NS3 protein expression compared to control conditions (**Fig 3D**). These observations suggest that RTN3L&S directly modulates the incorporation of replication viral material packages inside infectious HCV exosomes.

### Loading of replication-competent viral molecules inside exosomes is mediated by the C-terminal region of Reticulon-3

**Fig 4:**
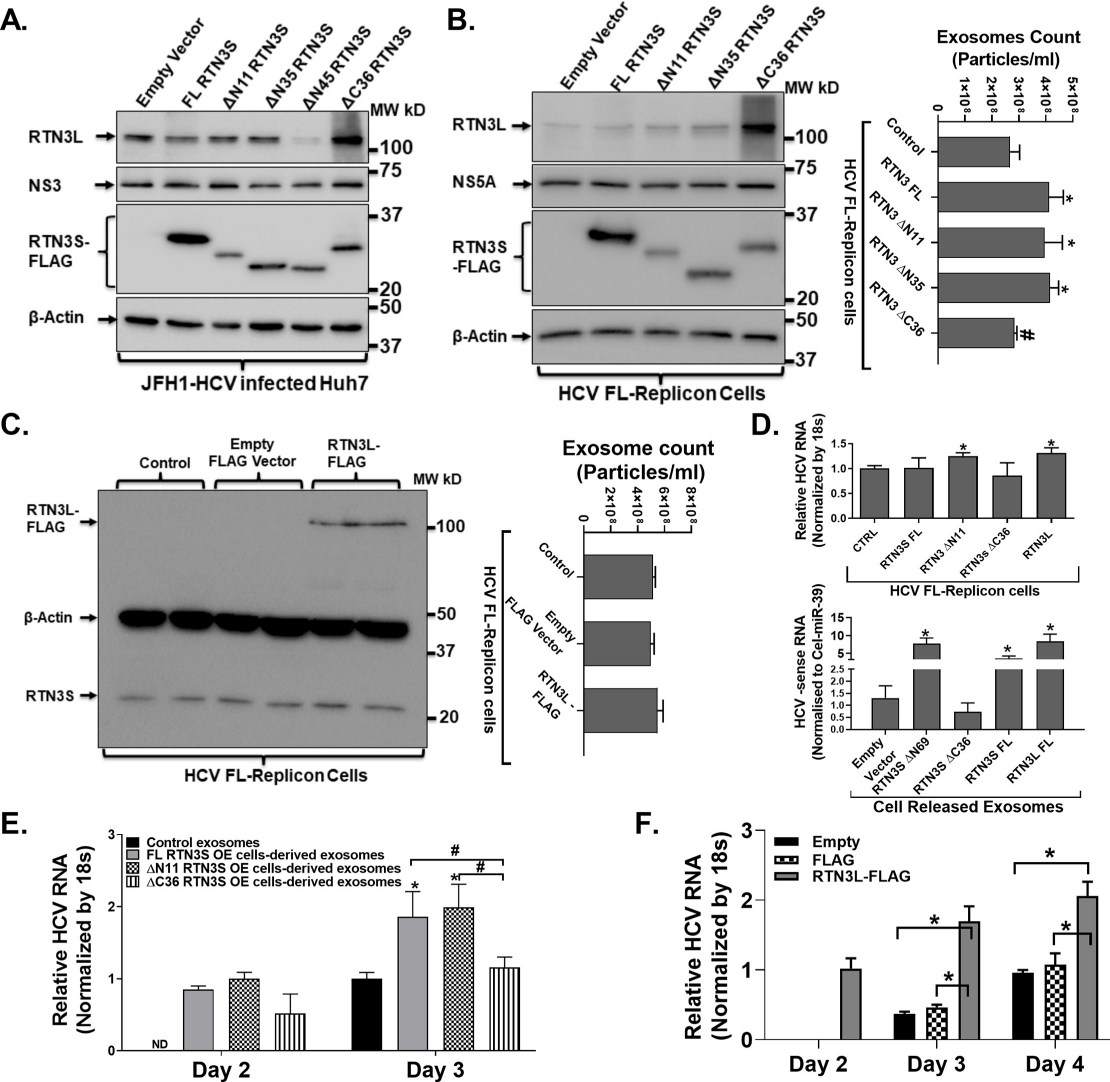
RTN3L&S carboxyl-terminal domain modulates the loading of replication-competent viral material inside infectious exosomes. (**A&B-Left panel**) Full-length Flag-tagged RTN3S, N-terminal deletion RTN3S, and C-terminal deletion mutants of RTN3 were overexpressed in HCV JFH-1 infected Huh7 and HCV FL-replicon cell. Total proteins were then extracted from cells 72h post-transfection and analyzed by western blotting probing antibodies targeting RTN3L, Flag, HCV NS3, and NS5A. β-actin served as a loading control. (**B-Right panel**) Cell released exosomes harvested from cell culture supernatants from HCV FL-replicon cells without and with the overexpression of full-length Flag-tagged RTN3S, N-terminal deletion RTN3S and C-terminal deletion mutants of RTN3 were quantified by NanoSight NS300. (**C**) Full-length Flag-tagged RTN3L were overexpressed in HCV FL-replicon cells. Total proteins were then extracted from cells 72h post-transfection and analyzed by western blotting probing antibodies targeting RTN3S and Flag. β-actin served as a loading control. (**D-Upper panel**) Total RNA was then extracted from cells for conditions described in B&C and quantified for HCV RNA using 18s RNA as a normalization control (**D-Lower Panel**) Total RNA was extracted from cell released exosomes from conditions described in (**B&C**) and analyzed for negative-sense HCV RNA by RT-qPCR using cel39 as an exogenous normalization control. (**E&F**) Cell released exosomes from conditions described in (**B&C**) were co-cultured with naïve Huh7 cells over a 3 to 4-days period. Total RNA was then extracted from cells and quantified for HCV RNA using 18s RNA as a normalization control. Data presented here are representative of 3 independently repeat experiments with */^**#**^p<0.05 was considered significant by Mann–Whitney U test.

Our transient knockdown experiment revealed that RTN3 might directly impact the loading of replication-competent viral exosomes (**Fig 3**). To further delineate this role, we additionally overexpressed wild type, N and C-terminal deletion mutants of RTNL&S in HCV infected cells alongside appropriate controls (**Fig 4A & 4B**). Our experiments revealed that over-expression of either the wild type or mutant isoforms in JFH1 infected Huh7 or HCV FL-replicon cells did not impact HCV replication as revealed by HCV NS3/NS5A western blotting analysis (**Fig 4A & 4B-Left panel**). Strikingly, overexpression of C-terminal deleted RTN3L&S mutants was associated with a corresponding increase in the native cellular forms of RTN3L&S proteins (**Fig 4A & 4B-Left panel**). However, we found that overexpression of the wild type or N-terminal deletion mutants of RTN3S (**Fig 4B-Right panel**) was associated with the increased cellular release of exosomes compared to empty vector-transfected control (**Fig 4B-Right panel**). Further, overexpression of C-terminal deletion mutants of RTN3 was associated with significantly reduced cell released exosomes compared to overexpression with full length and N-terminal deletion mutants (**Fig 4B-Right panel**). Overexpression of RTN3L did not impact RTN3S expression (**Fig 4C-Left panel**) or the number of cell-released exosomes (**Fig 4C-Right panel**). Given that cell release exosomes are loaded with replication viral RNA, we next assessed in the overexpression of RTN3L&S as well as mutant isoforms impacted the amount of viral RNA in HCV infected cells. While overexpression of RTN3S FL and RTN3S C36 mutants in FL-replicon cells did not impact HCV RNA levels, we found that the overexpression of RTN3SΔN11 and RTN3L was associated with a modest but statistically significant increase in cellular viral levels (**Fig 4D- Upper panel**). We however found that the over-expression of full-length RTN3S, RTN3SΔN11, and RTN3L in FL replicon cells significantly increased the enrichment of negative-sense HCV RNA in cell released exosomes. Remarkably, the over-expression of RTN3SΔC36 mutant in FL-replicon cells was associated with significantly reduced negative-sense HCV RNA enrichment in cell released exosomes (**Fig 4D- Lower panel**). Further, suggest that the overexpression of RTN3L in FL-replicon cells significantly increased HCV RNA in cells (**Fig 4D-upper panel**) and also increased the loading of viral RNA inside cell-released exosomes(**Fig 4D-Lower panel**). In addition, co-culture of exosomes from RTN3S wild type, N and C-terminal deletion mutants revealed that C-terminal mutants of RTN3 were less infectious as revealed by real-time qPCR (**Fig 4E**). These findings suggest that the C-terminal domain of RTN3 protein potentially mediates critical interactions and trafficking of replication-competent viral RNA and specific cellular host molecules for loading and release inside infectious viral exosomes. Finally, co-culture of cell released exosomes from RTN3L overexpressing cells revealed that these cell released exosomes were more infectious compared to exosomes from control conditions (**Fig 4F**)

### Treatment of HCV JFH-1 infected Huh7 cells reduced RTN3 expression and the number of cell-released infectious exosomes

**Fig 5:**
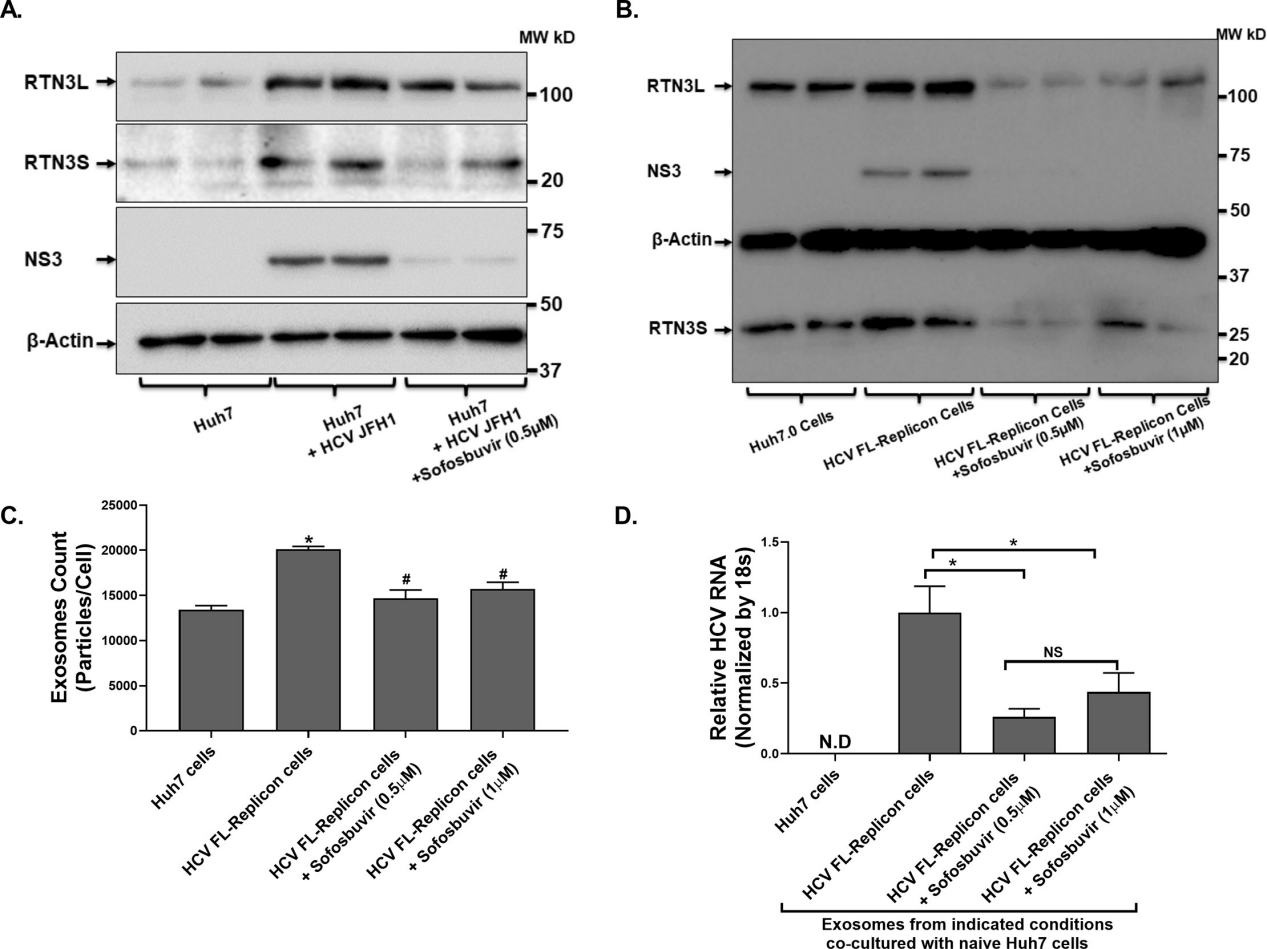
Treatment of HCV infected cells with Sofosbuvir significantly reduced HCV induced RTN3L&S and cell released infectious exosomes. (A) Huh7-JFH1 infected and (B) HCV FL-Replicon cells were treated with Sofosbuvir or not as indicated alongside non-treated Huh7.0 control cells. 10-days post-Sofosbuvir treatment, cell culture supernatant, and cells were harvested. (**A&B**) Total protein was extracted from cells and analyzed by western blotting probing for HCV NS3 and RTN3L&S with β-actin serving as a control for equal protein loading. (**C**) Cell released exosomes in cell-cultured supernatants were quantified by NanoSight NS300 and normalized per cell based on total cell counts at the end of treatment. (**D**) Cell released exosomes from treatment conditioning in (**B**) were co-cultured with naïve Huh7.0 cells (5000 Exosomes: 1 Huh7.0 cell) over 72h. Total RNA was then extracted from cells and analyzed by RT-qPCR for HCV RNA using 18s as a housekeeping gene. Data for 3 repeat experiments are presented with */^**#**^p<0.05 considered significant by the Mann -Whitney U test.

Hepatitis C can be treated by effective direct-acting antiviral agents. The impact of these treatments on cell released infectious exosomes remain ill-defined. Therefore, we treated HCV JFH1 infected Huh7 and HCV FL-replicon cells. Our western blotting analysis revealed that sofosbuvir (an NS5B polymerase inhibitor currently used several approved combination anti-HCV regimens) treatment of HCV infected cells abrogated viral replication (with reduced HCV NS3 expression) (**Fig 5A & 5B**). Further, the increased RTN3 expression, typically induced by HCV infection, was significantly decreased after sofosbuvir treatment (**Fig 5A & 5B**). Subsequently, the co-culture of exosomes from HCV infected treated cells with naïve Huh7 cells reduced both the number (**Fig 5C**) and infection capacity (**Fig 5D**) of exosomes compared to appropriate controls. These observations suggest that available HCV treatments, specifically sofosbuvir can directly suppress the release of infectious exosomes.

## Discussion

All viruses have developed sophisticated strategies to hijack host defense mechanisms using host cell molecules and pathways in unique ways to achieve effective infection. Several reports, including our studies, have revealed that HCV can hijack the host exosomal pathway to mediate HCV immunologic escape and persistence [11, 12]. However, the mechanism by which the HCV can modulate the specific loading of replication-competent viral material inside infectious exosomes remains undefined.

In the current study, we reveal that RTN3 protein can mediate the specific loading of replication-competent HCV RNA and specific cellular molecules for release inside infectious viral exosomes. We found that HCV JFH1 infection of Huh7 cells was associated with increased cellular expression of RTN3L&S protein isoforms. Similarly, western blot analysis revealed increased expression of RTN3L&S protein isoforms in liver samples from HCV infected individuals compared to healthy control (HCV negative) subjects. Further, knockdown and overexpression of RTN3 in HCV infected cells, respectively, decreased and increased the number of cells released infectious exosomes. Further, we demonstrate that RTN3L&S isoform proteins interacted with dsHCV RNA and that the c-terminal region is required for efficient loading of replication-competent viral material released inside infectious exosomes.

Reticulons are ER-located proteins (primary site for HCV replication) and despite their multiple isoforms, all share a common Reticulon homology domain (RHD) [23]. Reticulons have been shown to have differential effects on viral infections. In Flaviviruses, RNT3 was shown to be significantly enriched within membranous structural sites of virus replication [28, 43]. In the context of HCV infection, Lin et al revealed increased intrahepatic RTN3 levels which correlated with higher HCV genotype 1 RNA levels [37]. These observations are in concert with our findings which revealed increased RTN3L&S isoforms in HCV JFH1 infected Huh7 cells and liver samples from HCV infected treatment naïve individuals. Further, we revealed that cell released exosomes from in-vitro HCV infection and replication cell systems were enriched with RTN3L. Strikingly, serum exosomes from HCV infected individuals showed increased enrichment with RTN3L&S and HSP90 [11], which we revealed previously. There are reports that RTN3 can act as an anti-viral agent during HCV infection [29, 44]. Specifically, this study demonstrated that RTN3 interacts with HCV NS4B and negatively regulates viral replication [29]. The silencing of RTN3 in this study was associated with a significant increase in HCV replication which could be counteracted by the overexpression of recombinant RTN3 [29]. These observations are in contrast with our findings since we reveal here that knockdown or overexpression of RTN3S and RTN3ΔC36 did not impact HCV replication. However, some of our findings are in concert with reports by Tripathi *et al* who revealed that RTN3 knockdown did not impact HCV JFH1 replication [30]. Additionally, we found while overexpression of RTN3SΔN11 did not impact HCV NS3 protein in cells, it was associated with a modest but significant increase of cellular viral RNA. Together, our observations suggest that RTN3L&S knockdown and overexpression of wild type or the various isoform mutants might not impact viral gene translation but can certainly differentially impacts viral RNA turnover inside cells and the enrichment of viral RNA in cell released exosomes. Consequently, exosomes from RTN3 knockdown cells were less infectious compared to exosomes from released by RTN3 over-expressing HCV infected cells. Taken together, our findings support a notion that the various RTN3L&S isoforms perform different functions and can lead to opposite effects depending on their impact on viral replication and viral gene translation [29, 30].

For effective loading of infectious viral exosomes, efficient cellular trafficking and compartmentalization of specific molecules are required. As previously indicated, the biological function of RTN3 includes cellular trafficking most often involving its c-terminal domain interactions with other molecules [24]. The significance of this observation in the context of infectious exosome loading has not been exploited. Here, we show that RTN3 specifically differentially interacts with HCV dsRNA, miR-122 as well as in complex with HCV NS3 protein. Observed differential interactions of RTN3 with host and viral molecules in the JFH1 (HCV Genotype 2a) and FL-replicon (HCV Genotype 1b) infected cells can be attributed to the characteristic differences of the HCV strains, not to the parental Huh-7 cell line. Additionally, the trafficking and loading of exosomes involving RTN3 are most likely to be a very dynamic process and variabilities observed in our interaction experiments should not be uncommon. Further, we reveal that the wild type and N-terminal deletion mutants of RTN3L&S isoforms significantly induced the release of infectious viral exosomes. Strikingly, cellular overexpression of C-terminal deletion mutants of RTN3L&S resulted in a significant decrease in cell released infections exosomes. We also observed corresponding increased expression of RTN3L&S during cellular overexpression of c-terminal deletion mutants of RTN3L&S forms respectively. These novel observations suggest that the c-terminal domain of RTN3 plays an active role in the loading and generation of infectious viral exosomes. Our observation of corresponding increases in cellular RTN3L&S proteins following respective overexpression of C-terminal deletion mutants of RTN3L&S suggests possible compensatory regulation between both proteins to maintain infectious exosome generation. However, the cellular knockdown of both RTN3L&S did not completely block the cellular release of infectious viral exosomes. These observations suggest the existence of additional mechanisms that can mediate infectious exosome loading associated with HCV infection.

There is currently highly effective therapy for HCV infection, hence we assessed the effect of treatment on infection-induced increased RTN3 and infectious exosome release. We found that Sofosbuvir, an HCV NS5B nucleotide polymerase inhibitor [45], significantly reduced increased RTN3 expression caused by HCV infection. Furthermore, Sofosbuvir significantly reduced the cellular release of infectious viral exosomes. Our observation of increased RTN3L&S expression in liver and serum exosomes of HCV infected individuals, that decreased with anti-HCV treatment may have future applications in terms of complementary viral or disease prognostic biomarker. Further, in an era of effective DAA treatments for HCV infection, assessing exosomal or hepatic expression of RTN3 may serve to assess treatment outcomes and potential drug resistance.

**Fig 6:**
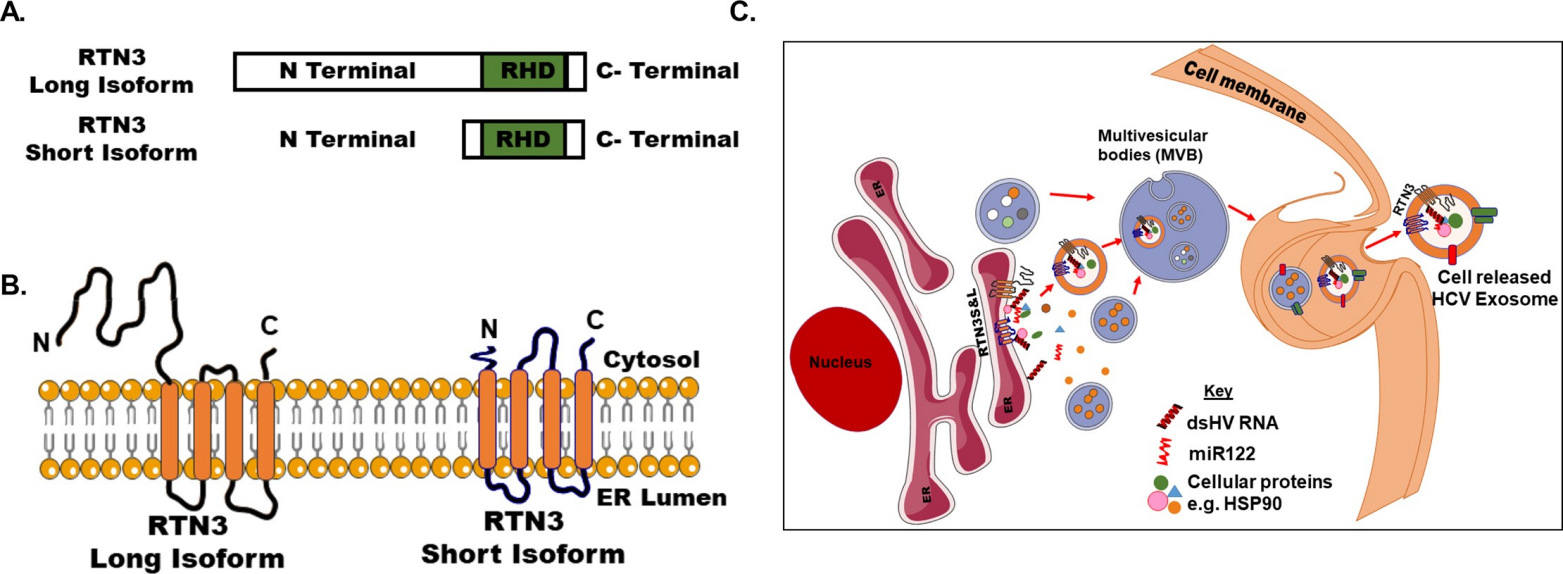
Schematics of RTN3 facilitates the loading of infectious HCV exosomes. (**A**) Schematic illustration of RTN3 long and short isoforms (RTN3&S) and (**B**) their cellular endoplasmic reticulum (ER) locations. (**C**) HCV infection is associated with increased expression of double-stranded (ds) HCV RNA in Huh7 cells. Double-stranded HCV RNA can interact with endoplasmic reticulum located RTN3L&S protein isoforms HCV infected Huh7 cells. Additionally, direct or indirect interactions also occur between other cellular proteins and nucleic acids with dsHCV RNA and RTN3L&S in HCV infectedHuh7 cells. RTN3L&S in HCV infected Huh7 cells can directly modulate the incorporation of replication-competent HCV dsRNA in association with other proteins inside membrane vesicles which are translocated to multivesicular bodies (MVB). This MVB in HCV infected Huh7 cells then fuse with the plasma membrane resulting in the release of infectious viral exosomes. Schematic illustration made use of some smart servier medical art templates (https://smart.servier.com).

In conclusion, our results provide evidence that RTN3 interacts with HCV NS3, replication-competent double-stranded HCV RNA, and miR-122. Reports have revealed increased endoplasmic reticulum (ER) blebbing in persistent HCV infected hepatocytes [46]. Taken together (**Fig 6**), we propose a model whereby HCV infection results in increased dsHCV RNA and specific cellular proteins interaction/binding to RTN3. Further, increased interactions/binding of dsHCV and specific cellular proteins coupled with cellular stress associated with chronic cellular infection results in ER blebbing to maintain cell fitness. This ER blebs containing dsHCV RNA and specific cellular proteins mature in multivesicular bodies and subsequently get released from cells as infectious exosomes. Given our novel revelations and the critical role of infectious exosomes in almost all viral infections, further clarification of the biological roles of RTNs in modulating the specific loading of pathogenic exosomes in the context of other viral infections should be evaluated.

## Supporting information

S1 Raw images (PDF)
