# Supplementary figures and images for: Reticulon-3 modulates the incorporation of replication competent hepatitis C virus molecules for release inside infectious exosomes
Source: PLoS One. 2020 Sep 17;15(9):e0239153. doi: 10.1371/journal.pone.0239153 (PMC7498005; doi:10.1371/journal.pone.0239153)

Figure 1

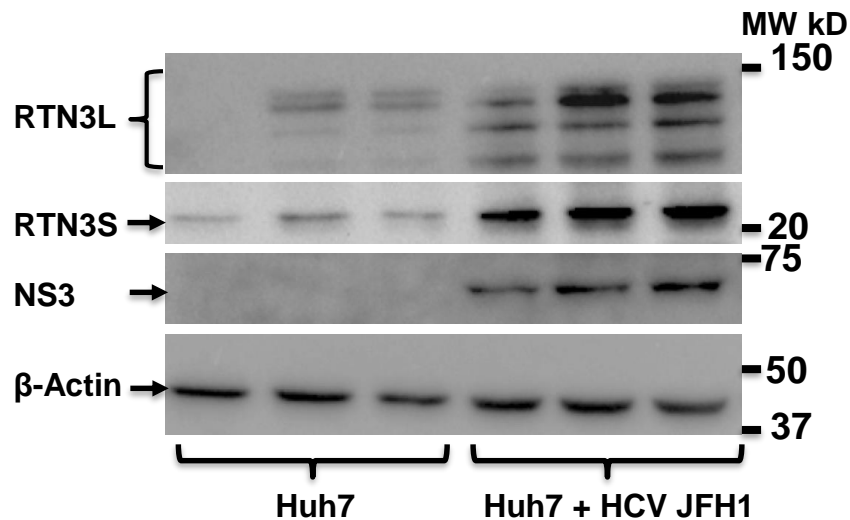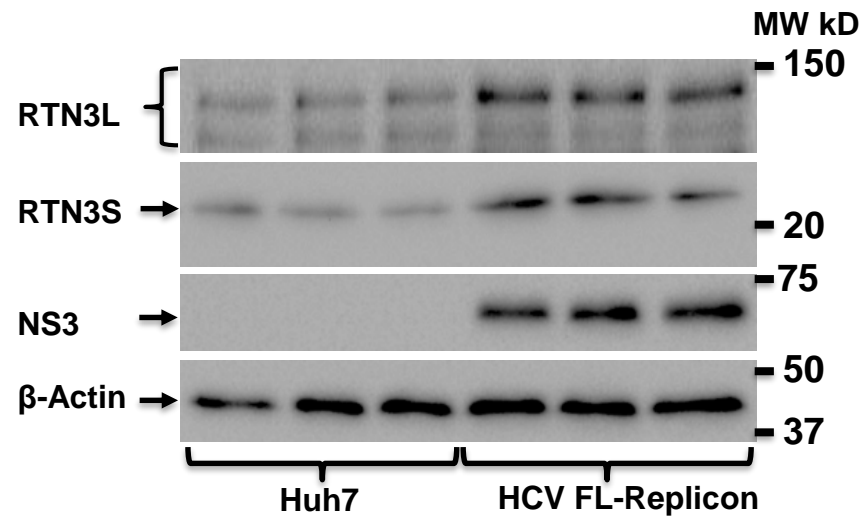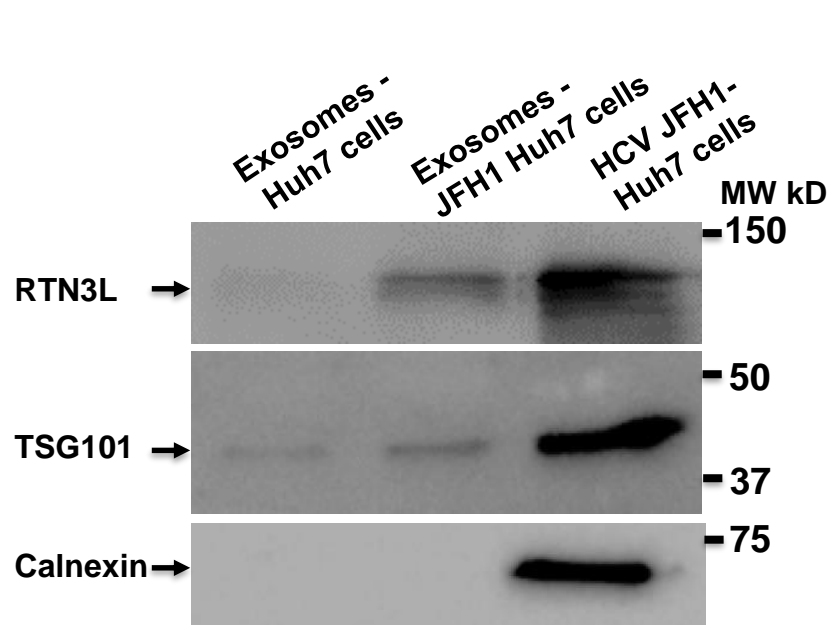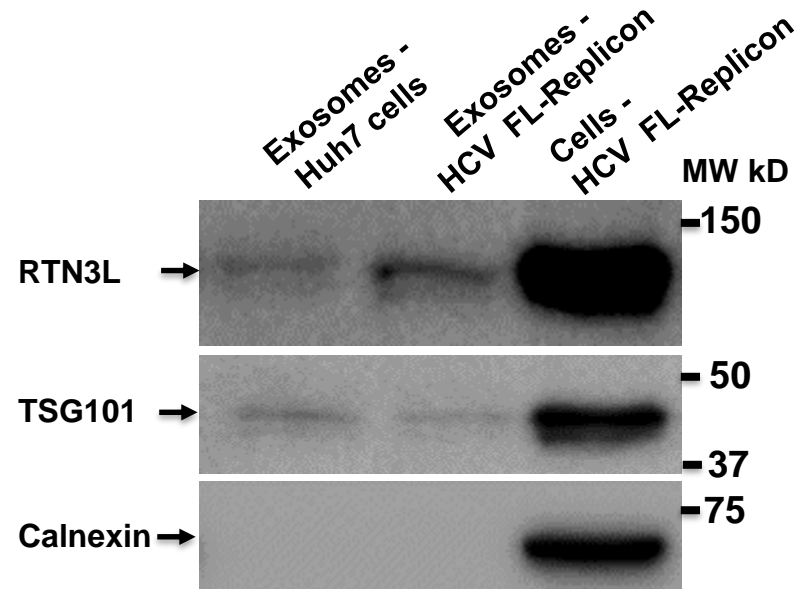

Figure 1

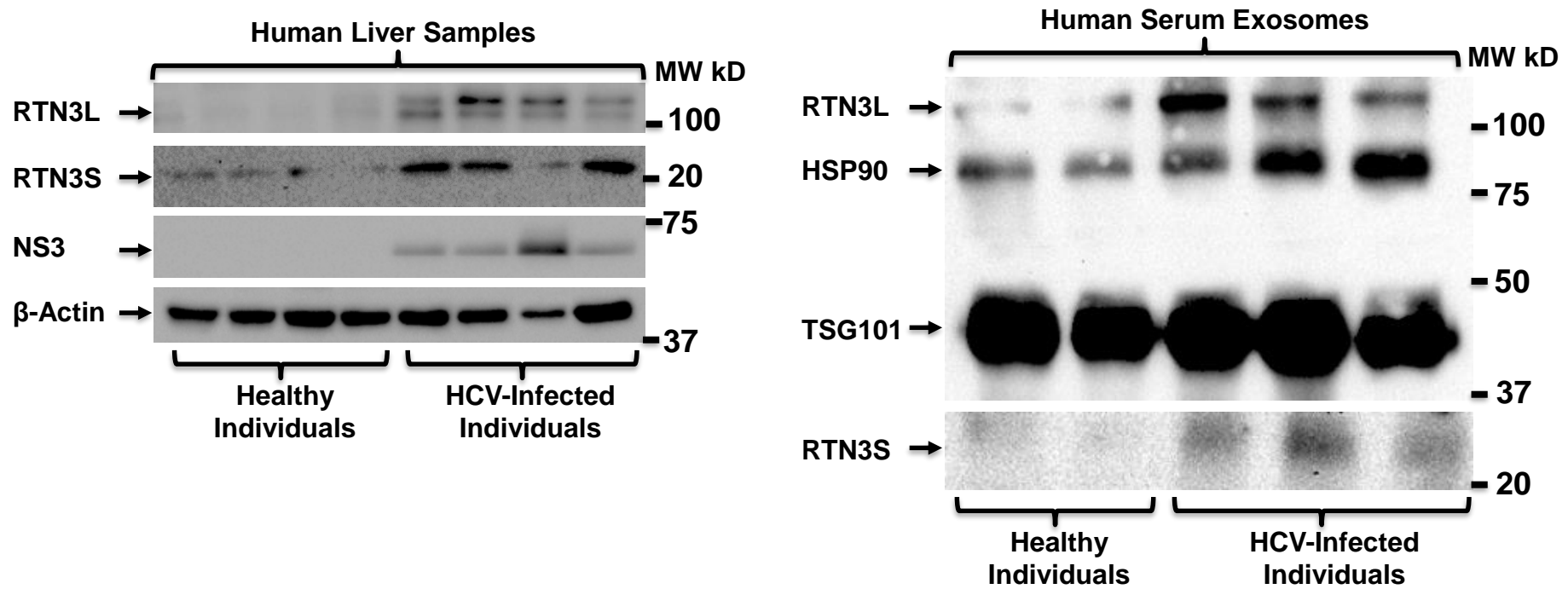

Figure 2

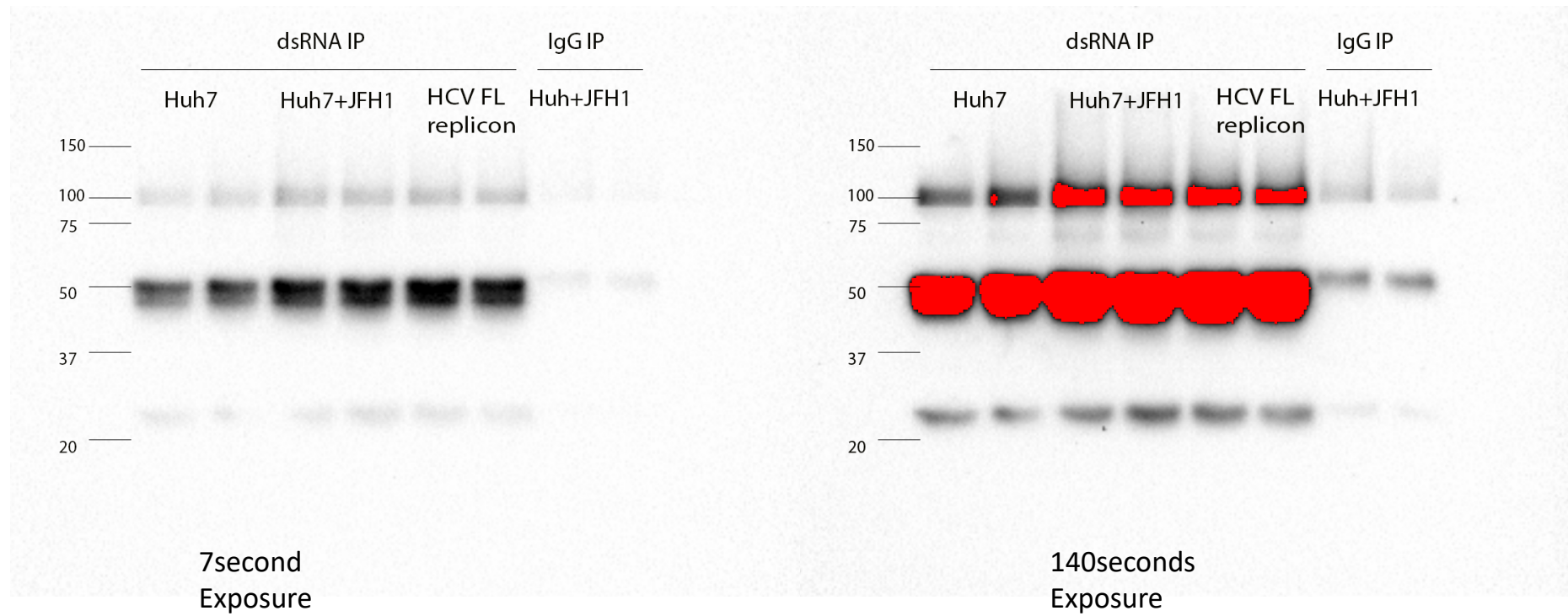

Figure 2- Actin Loading Control

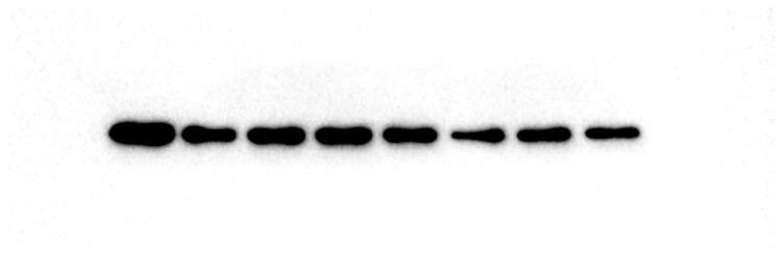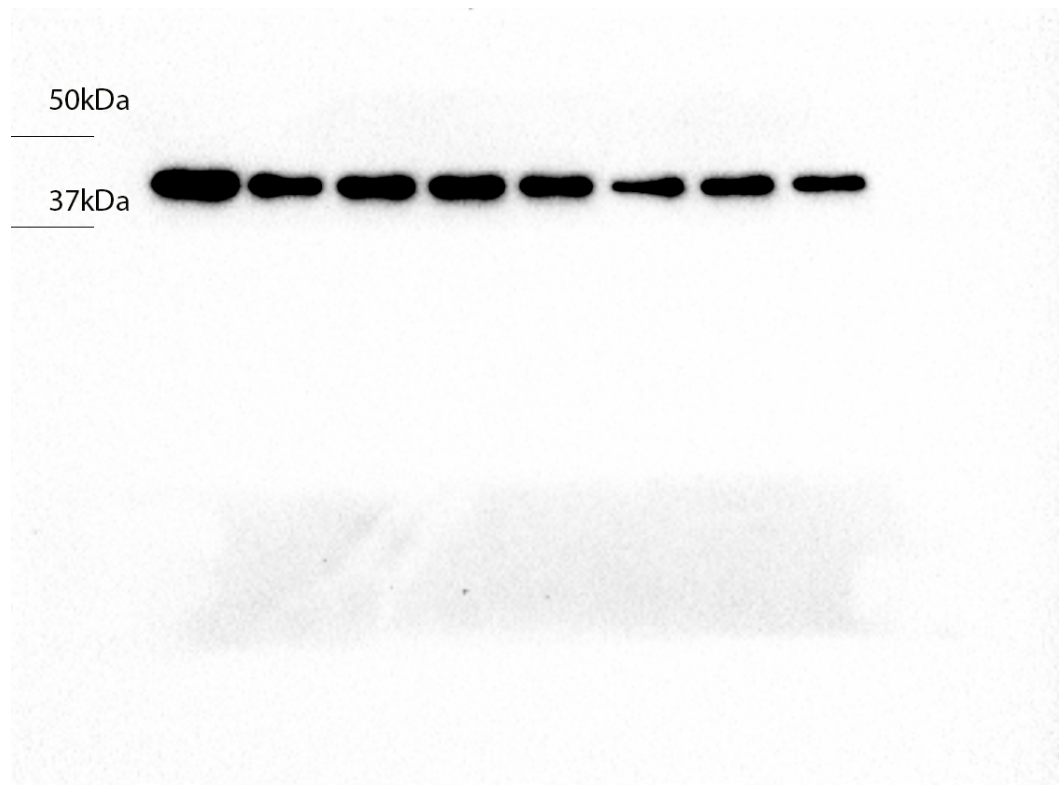

Figure 3

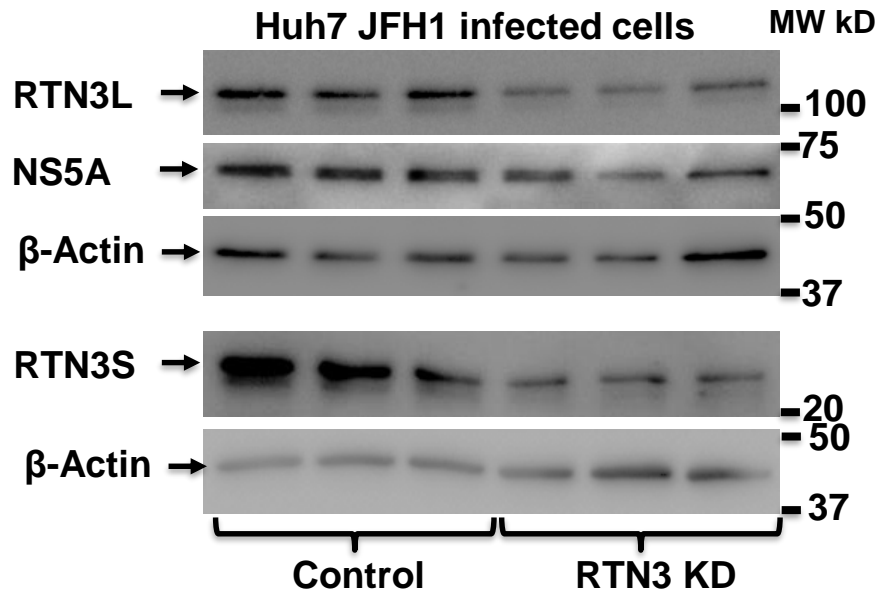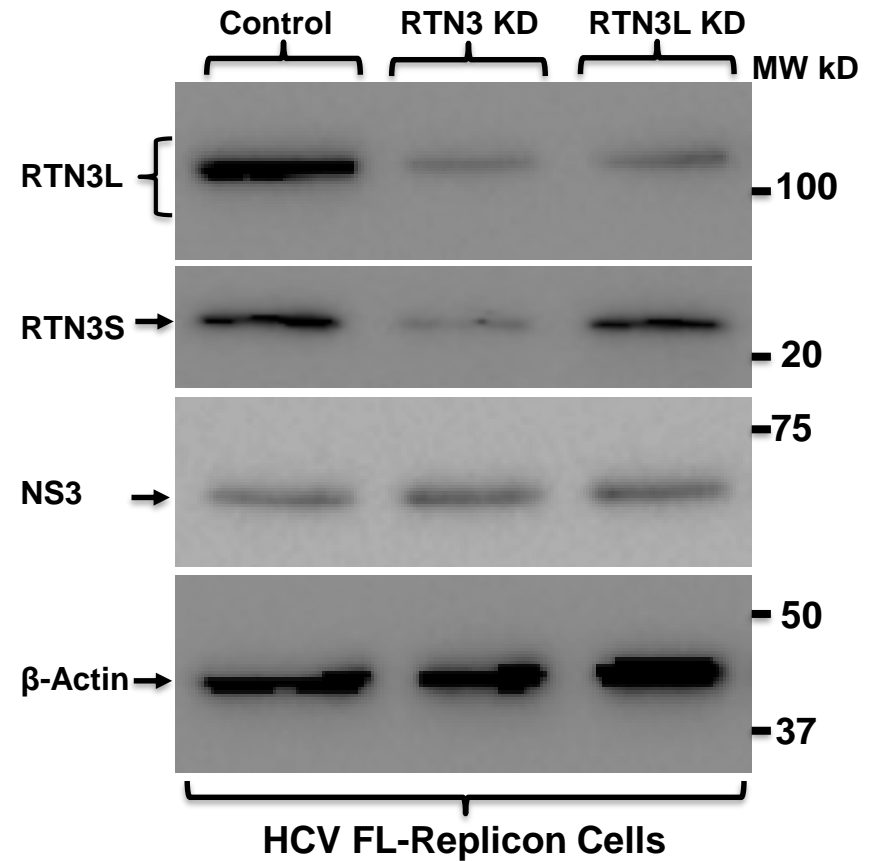

Figure 4

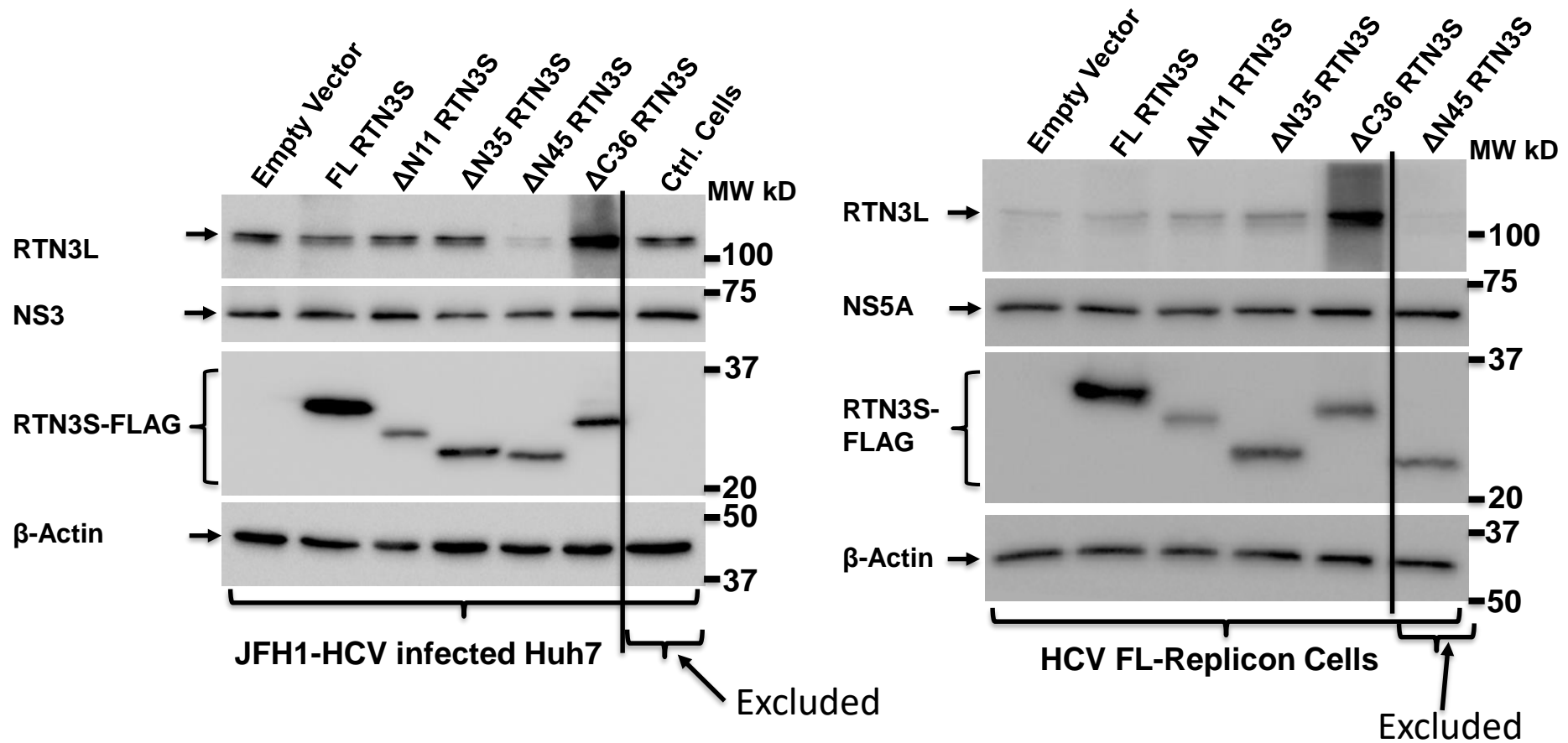

Figure 4

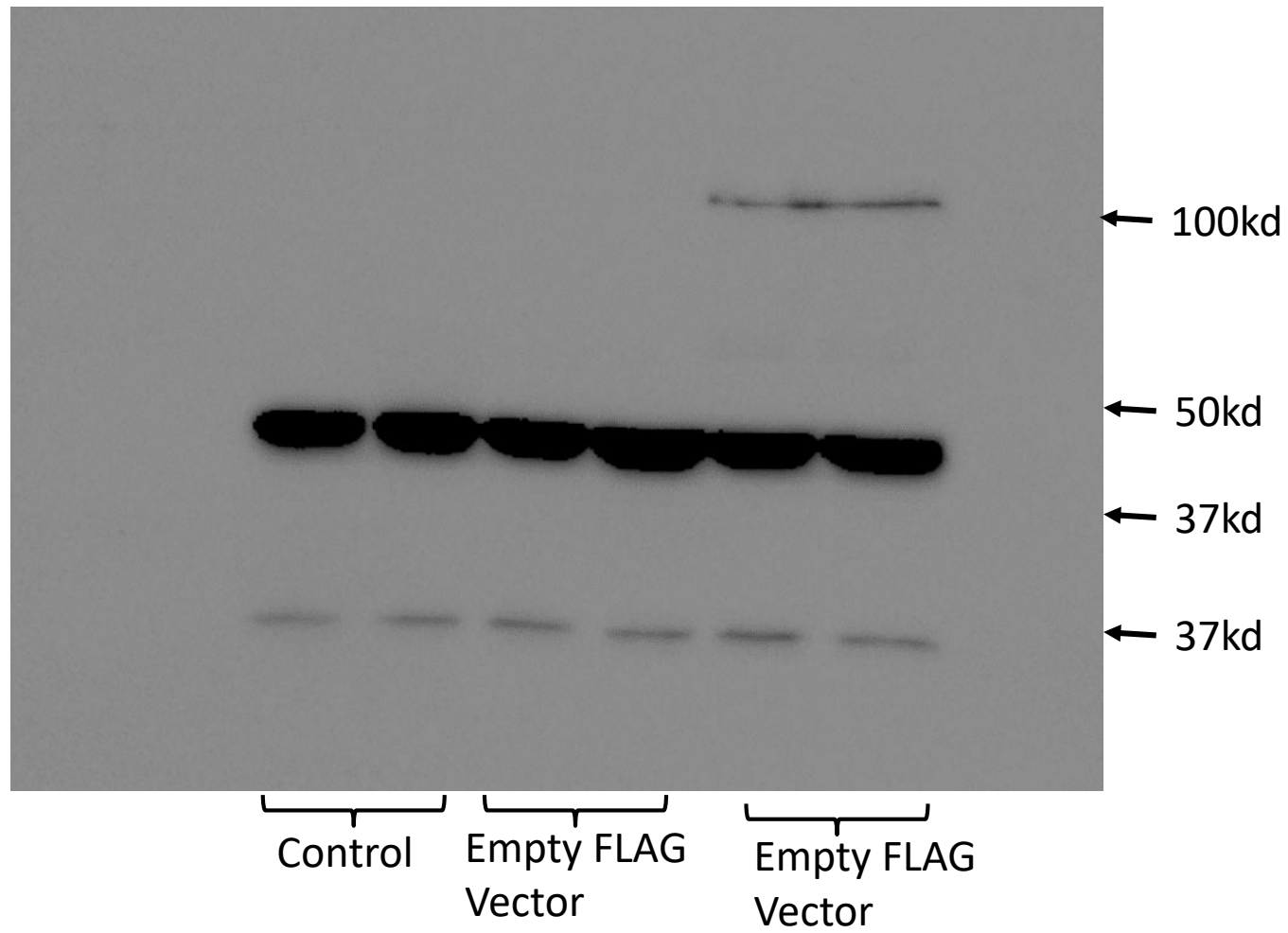

Figure 5

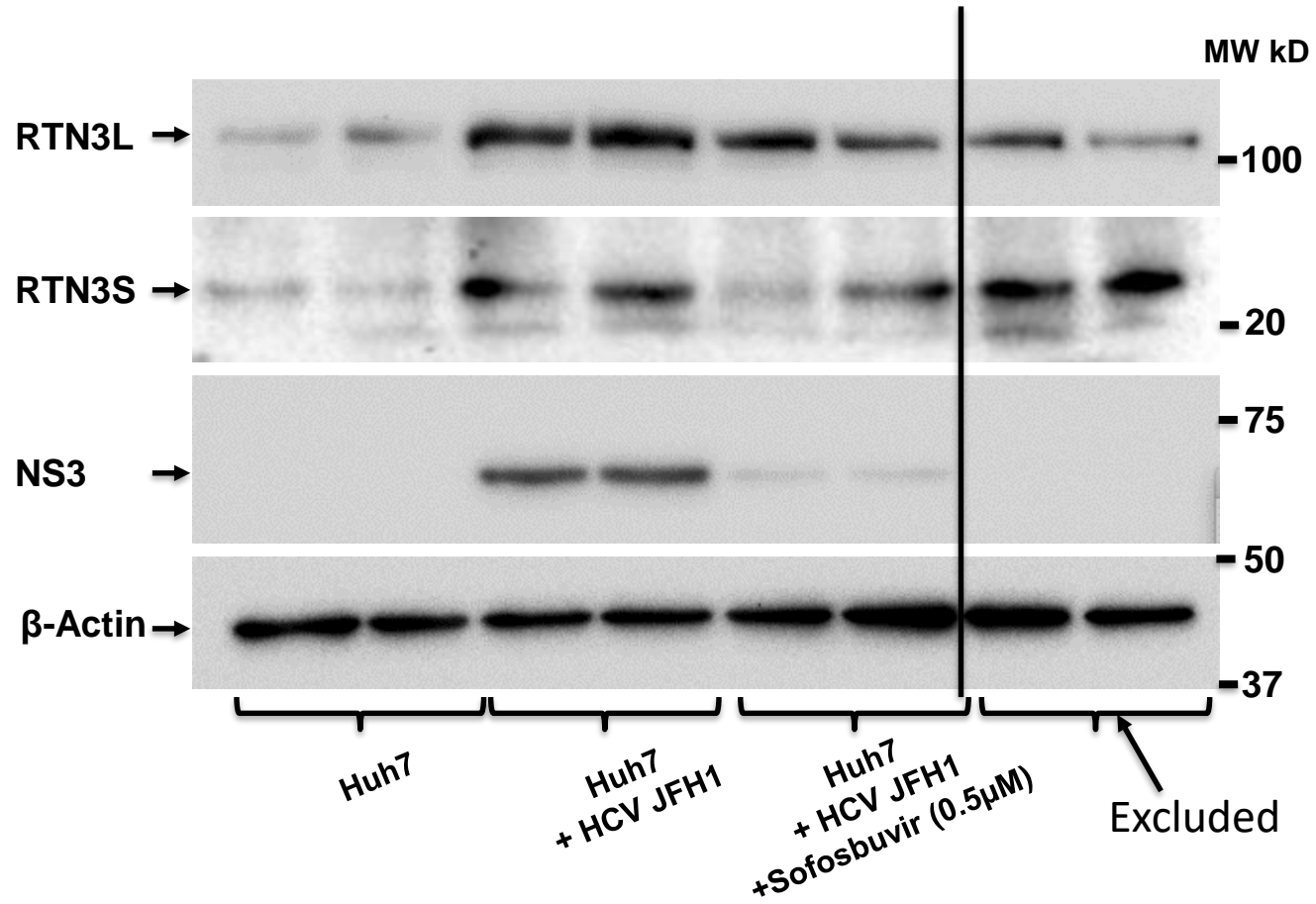

Figure 5

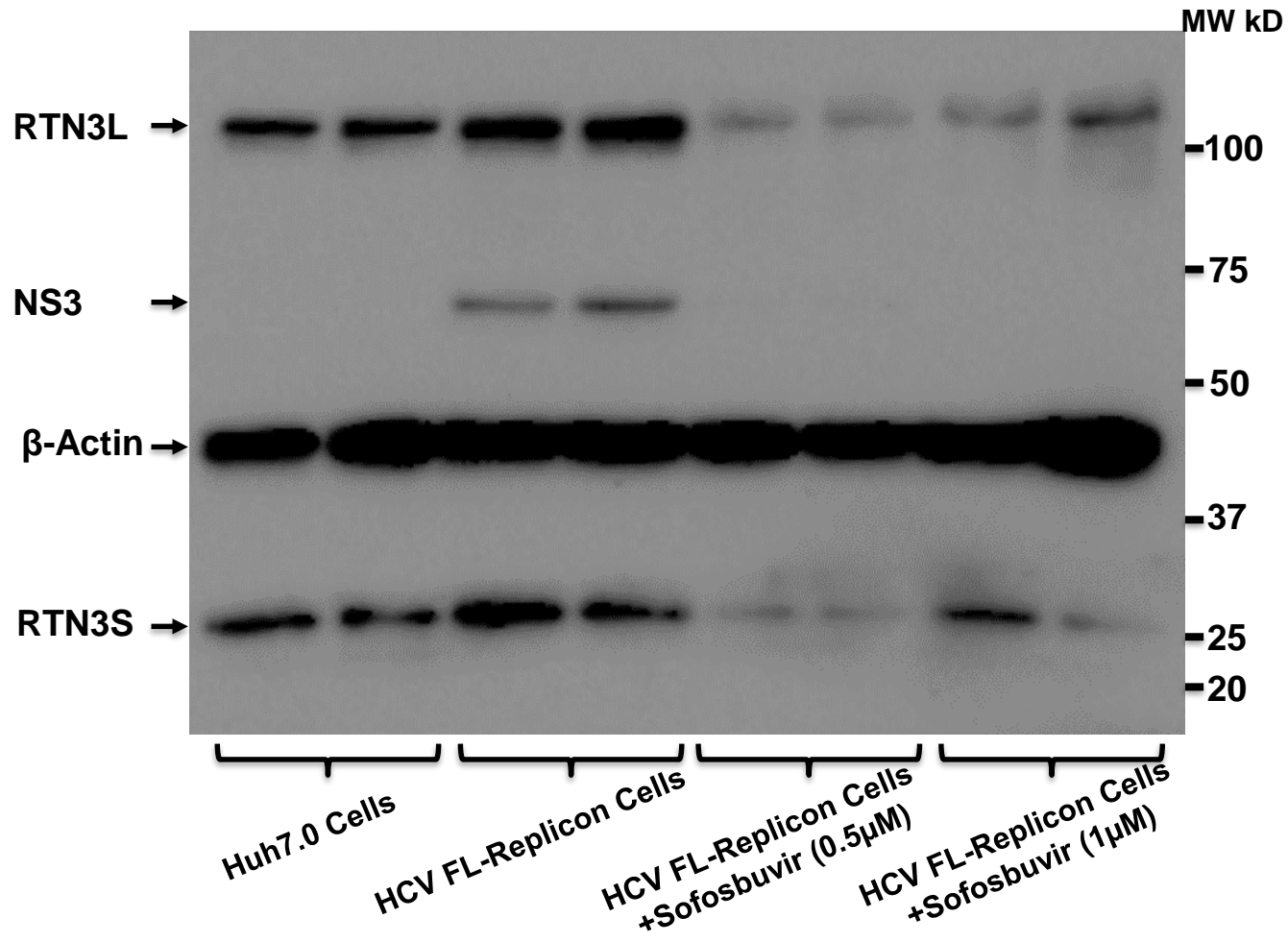

Figure 5

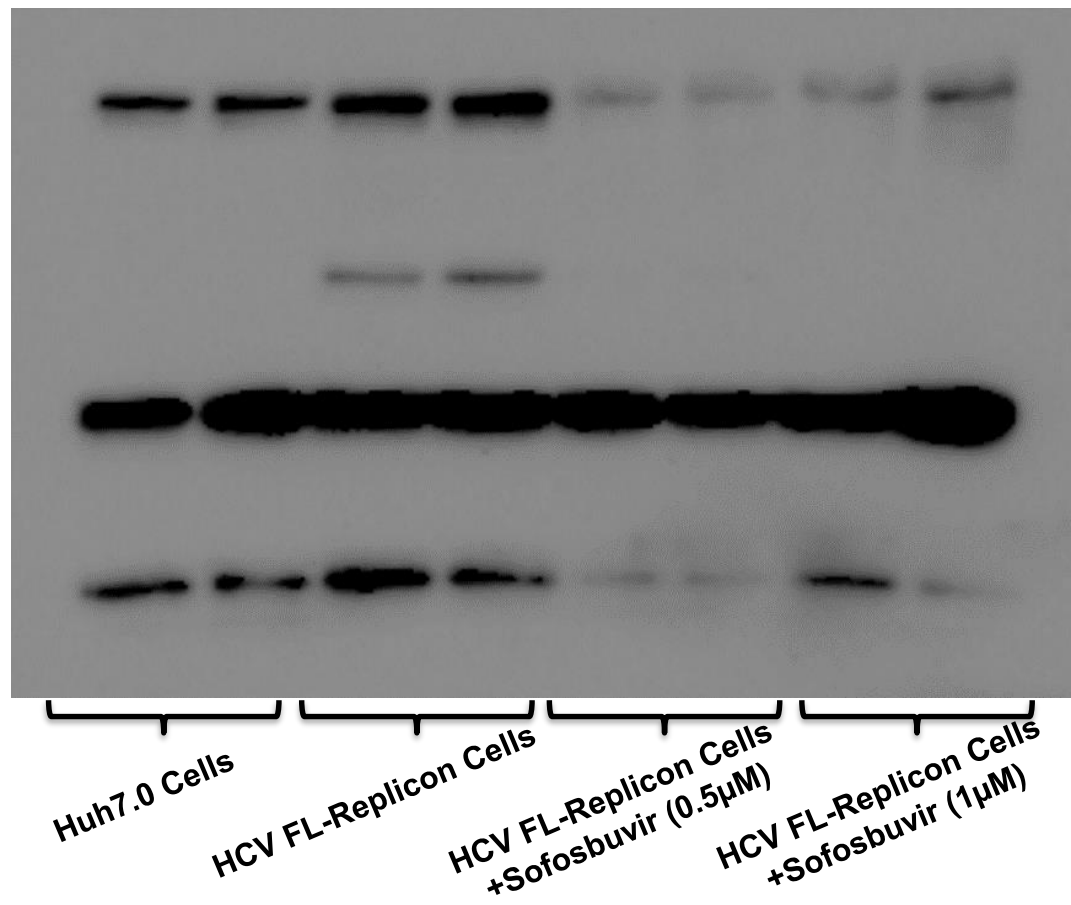

Supplement: S1 Raw images — (PDF) [file pone.0239153.s001.pdf]
